# Indirect Redox Transformations of Iron, Copper, and Chromium Catalyzed by Extremely Acidophilic Bacteria

**DOI:** 10.3389/fmicb.2017.00211

**Published:** 2017-02-10

**Authors:** D. Barrie Johnson, Sabrina Hedrich, Eva Pakostova

**Affiliations:** ^1^School of Biological Sciences, College of Natural Sciences, Bangor UniversityBangor, UK; ^2^Federal Institute for Geosciences and Natural ResourcesHannover, Germany

**Keywords:** acidophilic bacteria, chromium, copper, iron, oxido-reduction of metals, redox potentials

## Abstract

Experiments were carried out to examine redox transformations of copper and chromium by acidophilic bacteria (*Acidithiobacillus, Leptospirillum*, and *Acidiphilium*), and also of iron (III) reduction by *Acidithiobacillus* spp. under aerobic conditions. Reduction of iron (III) was found with all five species of *Acidithiobacillus* tested, grown aerobically on elemental sulfur. Cultures maintained at pH 1.0 for protracted periods displayed increasing propensity for aerobic iron (III) reduction, which was observed with cell-free culture liquors as well as those containing bacteria. *At. caldus* grown on hydrogen also reduced iron (III) under aerobic conditions, confirming that the unknown metabolite(s) responsible for iron (III) reduction were not (exclusively) sulfur intermediates. Reduction of copper (II) by aerobic cultures of sulfur-grown *Acidithiobacillus* spp. showed similar trends to iron (III) reduction in being more pronounced as culture pH declined, and occurring in both the presence and absence of cells. Cultures of *Acidithiobacillus* grown anaerobically on hydrogen only reduced copper (II) when iron (III) (which was also reduced) was also included; identical results were found with *Acidiphilium cryptum* grown micro-aerobically on glucose. Harvested biomass of hydrogen-grown *At. ferridurans* oxidized iron (II) but not copper (I), and copper (I) was only oxidized by growing cultures of *Acidithiobacillus* spp. when iron (II) was also included. The data confirmed that oxidation and reduction of copper were both mediated by acidophilic bacteria indirectly, via iron (II) and iron (III). No oxidation of chromium (III) by acidophilic bacteria was observed even when, in the case of *Leptospirillum* spp., the redox potential of oxidized cultures exceeded +900 mV. Cultures of *At. ferridurans* and *A. cryptum* reduced chromium (VI), though only when iron (III) was also present, confirming an indirect mechanism and contradicting an earlier report of direct chromium reduction by *A. cryptum*. Measurements of redox potentials of iron, copper and chromium couples in acidic, sulfate-containing liquors showed that these differed from situations where metals are not complexed by inorganic ligands, and supported the current observations of indirect copper oxido-reduction and chromium reduction mediated by acidophilic bacteria. The implications of these results for both industrial applications of acidophiles and for exobiology are discussed.

## Introduction

Acidophilic prokaryotes, defined as those that grow optimally at or below pH 3.0, display a far greater propensity for chemolithotrophy than other groups of bacteria and archaea that have higher pH growth optima (Johnson and Aguilera, 2015; Dopson, 2016). This is due to a number of factors, the most important of which is that their natural habitats are often rich in reduced sulfur and iron, and sulfide minerals, but often contain relatively small concentrations of dissolved organic carbon. In addition, the extreme acidity means that the solubility and bioavailability of cationic metals is much greater than in circum-neutral pH environments.

The most well studied chemolithotrophic life-styles (amongst acidophiles) are those based on the oxidation of reduced iron (Fe^2+^) by, for example, *Leptospirillum* spp. (Rawlings et al., 1999), and also of elemental sulfur and reduced inorganic sulfur compounds (RISCs) such as tetrathionate (S_4_O_6_^2-^) by *Acidithiobacillus* spp. and others (Dopson and Johnson, 2012). More recently, hydrogen has also been shown to be an electron donor for many species of acidophilic bacteria (Hedrich and Johnson, 2013a). In contrast, the range of electron acceptors used by extreme acidophiles appears to be more restricted than those used by neutrophilic prokaryotes, though dissimilatory reduction of iron (III) has been reported for many chemolithotrophic and heterotrophic acidophiles (Johnson et al., 2012). Redox transformations of inorganic electron donors and acceptors are frequently coupled in acidophile metabolisms. For example, both *Acidithiobacillus* (*At*.) *ferrooxidans* and *At. ferridurans* can couple the oxidation of elemental sulfur, RISCs and hydrogen to the reduction of molecular oxygen or iron (III).

In both natural and anthropogenic acidic environments, indigenous microorganisms encounter soluble transition metals (and metalloids, such as arsenic) that can exist in variable redox states. While the most abundant of these is almost invariably iron, other metals such as copper can be present in very elevated (>10 g/L) concentrations in pregnant leach solutions (PLS) generated in biomining operations, and in lower concentrations in mine drainage waters. The dissimilatory oxidation of iron (II) by acidophilic bacteria has been recognized since the early 1950s (Colmer et al., 1950). Iron reduction at low pH was first reported by Brock and Gustafson (1976), though the fact that dissimilatory reduction of iron (III) could be used to support the growth of some acidophiles (*At. ferrooxidans* and *Acidiphilium*) was only confirmed much later (Pronk and Johnson, 1992). In contrast to *Acidithiobacillus* spp., *Acidiphilium* spp. require trace amounts of oxygen for growth on iron (III), though non-growing cells can reduce iron in the absence of molecular oxygen. *Acidithiobacillus* spp. that can oxidize both iron (II) and reduced sulfur have a far greater propensity for coupling the oxidation of iron (II) to the reduction of molecular oxygen than for coupling the oxidation of sulfur either to oxygen (Sandoval Ponce et al., 2012) or to iron (III). In a report that appeared to contradict this principle, Sand (1989) noted that sulfur-grown *At. ferrooxidans* cultures that developed extremely low pH values (<1.3) became net productive of iron (II), even under aerobic conditions.

There have also been occasional reports of acidophilic bacteria catalyzing redox transformations of transition metals other than iron. For example, Nielsen and Beck (1972) found that *At. ferrooxidans* could grow on “museum-grade” chalcocite (Cu_2_S), and that Cu^2+^ was generated, while Lewis and Miller (1977) claimed that both Sn^2+^ and Cu^+^ could be oxidized by *At. ferrooxidans*, though neither could act as a sole energy source. Sugio et al. (1990) reported that an iron-oxidizing *Acidithiobacillus* sp. coupled the reduction of copper (II) to the oxidation of elemental sulfur, and suggested a direct (enzymatic) mechanism for the reaction with a pH optimum (5.0), which is well above pH optimum for growth of this acidophile. While there are no accounts of any acidophile being able to oxidize chromium (III), the reduction of chromium (VI) by three heterotrophic acidophiles, *Acidiphilium* (*A*.) *cryptum* (strain JF-5; Cummings et al., 2007) and *Acidocella aromatica*^T^ (Masaki et al., 2015), which are both mesophilic bacteria, and by a thermo-acidophilic archaeon (*Sulfolobus*; Masaki et al., 2016), has been described. Other reports of redox transformations of metals mediated by acidophilic prokaryotes include reduction of manganese (Sugio et al., 1988a), oxidation (Sugio et al., 1992) and reduction (Sugio et al., 1988b) of molybdenum, reduction of vanadium (Briand et al., 1996; Okibe et al., 2016) and oxidation of uranium (DiSpirito and Tuovinen, 1982).

Here we report the indirect dissimilatory redox transformations of three transition metals (iron, copper, and chromium) mediated by different genera and species of extremely acidophilic bacteria, *Acidithiobacillus, Leptospirillum*, and *Acidiphilium.*

## Materials and Methods

### Bacteria and Culture Conditions

Representative strains of five species of *Acidithiobacillus*, three of which (*At. ferrooxidans* (ATCC 23270^T^), *At. ferridurans* (ATCC 33020^T^) and *At. ferrivorans* (strain Peru6)) oxidize iron (II) as well as reduced sulfur, while two (*At. thiooxidans* DSM 14887^T^ and *At. caldus* DSM 8584^T^) do not oxidize iron, were used in experimental work. The Peru6 strain was used as this is the only isolate of *At. ferrivorans* that is known (like *At. ferrooxidans, At. ferridurans*, and *At. caldus*) to grow autotrophically on hydrogen. The iron (II)-oxidizing autotrophs *Leptospirillum* (*L*.) *ferrooxidans* (DSM 2705^T^) and *L. ferriphilum* (strain MT63), and the obligately heterotrophic iron (III)-reducer *A*. *cryptum* (strain SJH) were also used in some experiments. Physiological characteristics of the bacteria used are listed in **Table 1**.

**Table 1:** Some physiological characteristics of the acidophilic bacteria used in the present study.

Bacterium	Dissimilatory oxidation^∗^			Dissimilatory reduction^∗^	Temperature response^#^
	Fe(II)	S^0^	H_2_	Fe(III)	
*At. ferrooxidans*^T^	+	+	+	+	M
*At. ferridurans*^T^	+	+	+	+	M
*At. ferrivorans* Peru6	+	+	+	+	M
*At. thiooxidans*^T^	-	+	-	-	M
*At. caldus*^T^	-	+	+	-	MT
*L. ferrooxidans*^T^	+	-	-	-	M
*L. ferriphilum* MT63	+	-	-	-	MT
*A. cryptum* SJH	-	-	-	+	M

*^∗^supporting bacterial growth; ^#^M, mesophilic (*T*_*opt*_ 25–35°C); MT, moderately thermophilic (*T*_*opt*_ 40–50°C)*

Bacteria were grown in batch cultures in shake flasks and in pH- and temperature-controlled bioreactors (Electrolab, UK). Hydrogen-grown cultures were grown as described elsewhere (Hedrich and Johnson, 2013a) in sealed jars within which the atmosphere was enriched with both hydrogen and carbon dioxide. For anaerobic growth on hydrogen, oxygen was removed by including an AnaeroGen^TM^ sachet (Fisher, UK) in each sealed jar.

### Aerobic Reduction of Iron (III) by *Acidithiobacillus* spp.

Bacteria were grown in shake flask cultures (100 mL in 250 mL conical flasks) containing 0.5% (w/v) elemental sulfur “flower” (VWR, UK), 5 mM iron (III) sulfate, basal salts and trace elements (Ňancucheo et al., 2016) and adjusted initially to pH 3.0. Cultures were incubated at 30°C (*At. ferrooxidans, At. ferridurans* and *At. thiooxidans*) or 40°C (*At. caldus*), and samples withdrawn at regular intervals to measure pH, redox potentials (as *E*_H_ values) and concentrations of iron (II).

Following this, *At. ferridurans* and *At. caldus* were grown aerobically with elemental sulfur as electron donor in bioreactors maintained at 30 and 40°C, respectively, under controlled pH (*via* automated addition of acid or alkali). The bioreactors were aerated (1 L sterile air/min) and stirred at 100 rpm, and the growth medium used was as described for shake flask cultures, except that 50 μM iron (II) sulfate was used in place of 5 mM iron (III). The pH of the bioreactors was maintained at 2.0 (by automated addition of 1 M NaOH) until numbers of planktonic cells had reached ∼10^9^/mL, and then lowered, stepwise (using 1 M H_2_SO_4_), and held at pre-determined values, again by automated addition of 1 M NaOH, ultimately to pH 1.0. Samples were withdrawn at regular intervals and their potential for reducing iron (III) assessed. This involved adding iron (III) sulfate (1 mM, final concentration) to 5 mL of culture (maintained aerobically), and determining concentrations of iron (II) after 5 min, and again after 2 and 5 h. During the period when the bioreactors were held at pH 1.0, iron (III) reduction was also assessed using cell-free culture liquor samples (obtained by centrifuging samples at 10,000 × *g* for 5 min) and values compared with those using samples that contained bacteria.

To determine whether the potential for iron (III) reduction was confined to cultures grown on sulfur, *At. caldus* was grown aerobically with hydrogen as sole electron donor in culture media adjusted (with sulfuric acid) to either pH 1.0 or 2.0. These were incubated for up to 20 days, and samples withdrawn periodically to measure culture optical densities (at 600 nm, as a measure of growth) and to determine the iron (III)-reducing potential of whole culture and cell-free samples, as described above.

### Redox Transformations of Copper by Acidophilic Bacteria

Prior to testing cultures for their abilities to catalyze the dissimilatory reduction of copper (II) or oxidation of copper (I) (or both), minimum inhibitory concentrations (MICs) of copper (I) were determined for the various acidophile species used. For this, solutions of copper (II) sulfate (pH 2.0) were reduced by adding different volumes of 1 M hydroxylamine hydrochloride to the culture media. In each case, copper (II) was present in excess in order to ensure that there was no residual hydroxylamine which might otherwise have compromised the results obtained (hydroxylamine decomposes to nitrous oxide and water upon reaction with copper (II)). Cultures were set up with *At. ferrooxidans* and *At. ferridurans* (both pre-grown on hydrogen), *L. ferrooxidans* and *L. ferriphilum* (grown on iron, using iron (III)-free washed cell suspensions), and glucose-grown *A. cryptum*. Iron (II) or glucose was provided as electron donor, and growth was confirmed from cell counts and/or monitoring iron (II) oxidation.

Shake flask cultures, similar to those described for iron reduction except that iron (III) sulfate was replaced by 5 mM copper (II) sulfate and 100 μM iron (II) sulfate added to provide nutritional amounts of iron, were set up to examine whether copper (II) was reduced by *Acidithiobacillus* spp. when oxidizing elemental sulfur and incubated under aerobic conditions. In addition, samples of cultures of *At. caldus* and *At. ferridurans* grown aerobically on sulfur in pH-controlled bioreactors, as described above, were tested occasionally for their abilities to reduce copper (II).

Copper (II) reduction by autotrophic acidophiles was tested in cultures grown with hydrogen as electron donor, under anoxic conditions. This eliminated the possibility that changes in copper speciation was mediated by reduced sulfur compounds (e.g., copper (II) is well known to be reduced, and copper (I) to be complexed, by thiosulfate). *At. ferrooxidans* and *At. ferridurans* were grown in liquid media (pH 2.0) containing different concentrations of copper (II) sulfate and iron (III) sulfate (or 50 μM iron (II) sulfate, as control cultures) and incubated, anaerobically, under hydrogen-enriched atmospheres. Samples were removed at intervals to measure concentrations of copper (I) and iron (III). *A. cryptum* was grown (with glucose as electron donor) under both micro-aerobic and anaerobic conditions in a liquid medium (pH 2.3), again containing varying concentrations of copper (II) sulfate and iron (III) sulfate and changes in copper and iron speciation and redox potentials recorded. Growth and reduction of copper (II) was also tested using *At. caldus* and *At. ferrivorans*, incubated anaerobically with hydrogen as sole electron acceptor.

The ability of resting cells of *At. ferridurans* to catalyze the dissimilatory oxidation of copper (I) was tested by first growing a culture aerobically (at pH 2.0) with hydrogen as sole electron donor, to a cell density of ∼10^9^/mL, harvesting and re-suspending cells in basal salts (pH 2.0). A copper (I) solution was prepared by reducing a solution of copper (II) sulfate with hydroxylamine hydrochloride (1 M), again ensuring an excess of copper (II) to avoid any carryover of non-oxidized hydroxylamine. The cell suspension and copper (I) solutions were mixed to give a concentration of about 3 mM copper (I), and incubated at 30°C for 2 h, during which time samples were removed at regular intervals to determine residual concentrations of copper (I). Two controls were run in parallel: (i) non-inoculated copper (I) + pH 2.0 basal salts; (ii) an inoculated control containing 5 mM iron (II) as well as copper (I), which was monitored for concentrations of reduced iron as well as copper (I).

To determine whether oxidation of copper (I) occurred in growing cultures of iron-oxidizing acidophiles, an experiment was set up using media containing copper (I), both with and without added iron (II). Copper (II) sulfate was added (to 5 mM) to basal salts/trace elements liquid medium (pH 2.0) and hydroxylamine hydrochloride (as a sterile 1 M solution) added to reduce ∼60% of the copper present to copper (I). Iron (II) sulfate was added to 50% of the cultures (to 5 mM) which were then inoculated with either *At. ferrooxidans* or *At. ferridurans*, both grown on hydrogen as electron donor. Flasks were incubated, shaken, at 30°C, and samples withdrawn at regular intervals to measure concentrations of copper (I) and iron (II), and redox potentials. To examine the stability of copper (I) in non-inoculated media, separate shake flasks [amended, or not, with 5 mM iron (II) sulfate] were incubated alongside those containing the acidithiobacilli.

### Redox Transformations of Chromium by Acidophilic Bacteria

As with copper, the relative toxicities of both reduced (Cr^3+^; supplied as Cr_2_(SO_4_)_3_) and oxidized (CrO_4_^2-^; supplied as Na_2_CrO_4_) chromium were determined for cultures grown aerobically on hydrogen (*At. ferrooxidans, At. ferridurans*, and *At. ferrivorans*) at pH 2.0, or on glucose (*A. cryptum*) at pH 2.5, prior to testing for dissimilatory oxidation and reduction of the metal. The growth media used contained 50 μM iron (III) sulfate in order to avoid abiotic reduction of chromium (VI) by iron (II). The tolerance of *L. ferrooxidans* and *L. ferriphilum* was tested only for chromium (III), as both of these bacteria use only iron (II) as electron donor. Growth of cultures was confirmed from monitoring changes in cell numbers and (where appropriate) iron (II) oxidation.

Chromium (III) oxidation was tested (by iron-oxidizing acidophiles only) using both growing and resting cultures. For the former, liquid media containing 10 mM chromium (III) and either 0 or 10 mM iron (II) (pH 2.0) were inoculated with active cultures of *At. ferrooxidans, At. ferridurans, At. ferrivorans, L. ferrooxidans*, and *L. ferriphilum*, and changes in concentrations of iron (II) and chromium (VI) monitored for up to 18 days. Oxidation of chromium (III) by fully oxidized cultures of the same iron-oxidizing acidophiles was tested by growing cultures aerobically in a 20 mM iron (II) sulfate medium (initial pH 1.8) to completion of oxidation (determined by the maximum *E*_H_ values measured), adding 10 mM chromium (III) sulfate and measuring changes in *E*_H_ values and chromium (VI) concentrations after 5 min.

Reduction of chromium (VI) was also tested using both growing and resting cultures of *At. ferrooxidans, At. ferridurans*, and *At. ferrivorans*. Bacteria were grown anaerobically with hydrogen as electron donor in media containing iron (III) (125 μM or 5 mM) with or without 25 μM chromium (VI) and concentrations of iron (II) and chromium (VI) measured after 10 days. Chromium (VI) was added (to 200 μM) to chromium-free cultures of *At. ferridurans* grown anaerobically on hydrogen and iron (III), and residual concentrations of chromium (VI) and iron (II) measured after 5 min. To test chromium (VI) reduction by non-growing cultures, the same three *Acidithiobacillus* spp. were grown aerobically (again with hydrogen as electron donor) in chromium-free media. Cultures (containing >10^9^ bacteria/mL) were harvested, washed and re-suspended in basal salts (pH 2.0). To aliquots of each of these was added either 25 μM chromium (VI), 1 mM iron (III), or 25 μM chromium (VI) + 1 mM iron (III). Cell suspensions were sparged (at 30°C) with either pure N_2_ or H_2_/N_2_ for up to 2 h, and changes in concentrations of chromium (VI) and iron (II) recorded.

Chromium (VI) reduction by growing cultures and cell suspensions of *A. cryptum* was also tested. Chromium (VI) (100 μM – 1 mM) was added to cultures grown under micro-aerobic conditions in which all of the iron (III) present initially (4.6 mM) had been reduced to iron (II), and chromium (VI) concentrations measured after 5 min. Chromium (VI) reduction by harvested biomass of *A. cryptum* used a similar protocol to that described above for the *Acidithiobacillus* spp. except that biomass was grown aerobically at pH 2.5 with glucose as electron donor, and reduction by cell suspensions examined under both anaerobic (N_2_-sparged) and “micro-aerobic” conditions (10 mL of suspension in non-gassed 25 mL bottles) both in the presence and absence of added glucose (1 mM), at 30°C.

### Measurements of Standard Redox Potentials in Acidic, Sulfate-Containing Solutions

The standard redox potentials of the iron (II)/iron(III), copper (I)/copper(II), and chromium (III)/chromium (VI) couples were determined experimentally in defined acidic, sulfate-containing solutions. For the iron couple, equimolar (10 mM) solutions of iron (II) and iron (III) (as sulfate salts) were prepared and redox potentials measured at between pH 0.495 and 2.25. The effect on redox potentials of adding 50 or 100 mM magnesium sulfate to the iron (II)/iron (III) solution at pH 2.25 was also recorded. For copper, a 5 mM copper (II) sulfate solution (pH 2.0) was partially reduced by adding different values of 1 M hydroxylamine hydrochloride, and copper (I) concentrations determined. The redox potentials of three solutions containing varying ratios of copper (I)/copper (II) were measured, and the *E*_H_^0^ determined in each case, using the Nernst equation. In the case of chromium, an acidic (pH 2.43) solution containing equimolar concentrations of chromium (III) sulfate and sodium chromate was prepared and the redox potential (*E*_H_^0^) measured. The solution was then progressively acidified (with H_2_SO_4_) to pH 1.78, and the response to this on solution *E*_H_^0^ values recorded.

### Solubility and Stability of Copper (I) in Acidic Iron (II) Sulfate Solutions

The solubility of copper (I) chloride (Alfa Aesar, Ward Hill, MA, USA) in acidic (pH 2.0) water and in solutions of iron (II) sulfate (10 mM to 1 M, also at pH 2.0) was determined. The redox potentials of these solutions were also measured immediately after being prepared. The solutions were then left at room temperature (*ca*. 22°C) for up to 24 h, when they were visually inspected and tested for concentrations of copper (I) and copper (II), and redox potentials measured.

### Analytical Techniques

Concentrations of iron (II) were determined using the Ferrozine colorimetric assay (Stookey, 1970). Total iron was measured using the same assay after reducing soluble iron (III) to iron (II) with ascorbic acid, and iron (III) concentrations from differences in total and iron (II) concentrations. Concentrations of copper (I) were determined using the bicinchoninic acid colorimetric assay (Anwar et al., 2000), total copper following reduction of copper (II) to copper (I) with hydroxylamine, and copper (II) determined from differences in total copper and copper (I) concentrations. Concentrations of chromium (VI) were measured by ion chromatography (Phesatcha et al., 2012), using a Dionex DX-320 ion chromatograph attached to an Ion Pac CS5A column and an AD25 absorbance detector (Dionex, Sunnyvale, CA, USA), and analyzed using Chromeleon software (version 6.40) (Ňancucheo and Johnson, 2012).

A pHase combination glass electrode (VWR International, UK) was used to measure pH values, and a combined platinum Pt sensing electrode and a Ag/AgCl reference electrode (Thermo Fisher Scientific Inc., USA) to measure redox potentials, which were adjusted to be relative to a standard hydrogen electrode (*E*_H_ values). The redox electrode was standardized against two reference solutions of known redox potentials (ZoBell’s and Light’s). Both electrodes were used in conjunction with an Accumet 50 pH/redox meter.

## Results

### Aerobic Reduction of Iron (III) by *Acidithiobacillus* spp.

**FIGURE 1:**
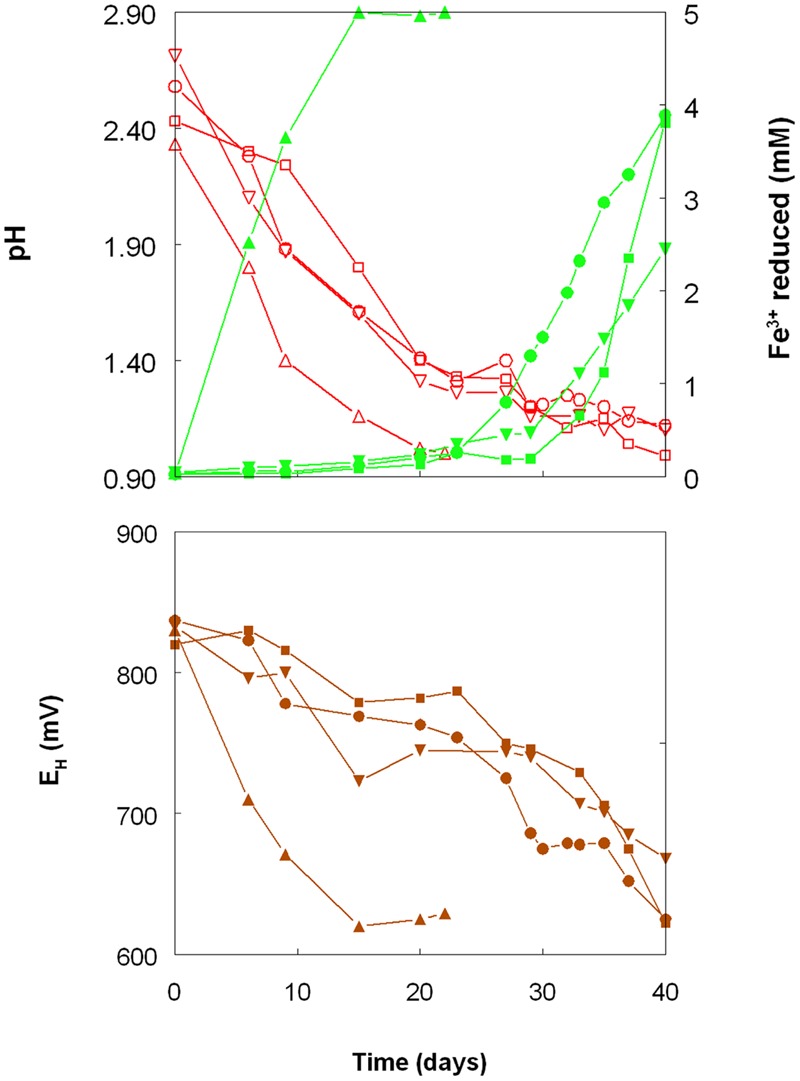
**Changes in pH values (red lines), amounts of iron (III) reduced (green lines) and redox potentials (***E***^**H**^; brown lines) in shake flask cultures of ***Acidithiobacillus*** spp. grown aerobically on elemental sulfur in the presence of 5 mM ferric iron.** Key: (●, ○) *Acidithiobacillus ferrooxidans*^T^; (■, □) *At. ferridurans*^T^; (▲, △) *At. caldus*^T^; (▼, ∇); *At. thiooxidans*^T^.

As shown in **Figure 1**, the pH of shake flask cultures of *Acidithiobacillus* spp. grown aerobically on elemental sulfur declined as incubation progressed, due to the production of sulfuric acid:

$$2S^{0}+2H_{2}O+3O_{2}\to 3H^{+}+{\mathrm{HSO}}_{4}^{-}+{\mathrm{SO}}_{4}{{}^{2}}^{-}.$$

The rates of acid production were similar in cultures of the three mesophilic species, but faster with those of the moderate thermophile *At. caldus*. Corresponding trends were also noted with iron (III) reduction and, consequentially, decreases in culture *E*_H_ values. Iron (III) was reduced to iron (II) in aerobic cultures of *At. caldus* from the start of the experiment, and was complete by day 12. In contrast, concentrations of iron (II) increased slowly in cultures of the three mesophilic species up to ∼day 25 (when pH values had fallen to ∼1.3) at which point the increases became much more rapid.

**FIGURE 2:**
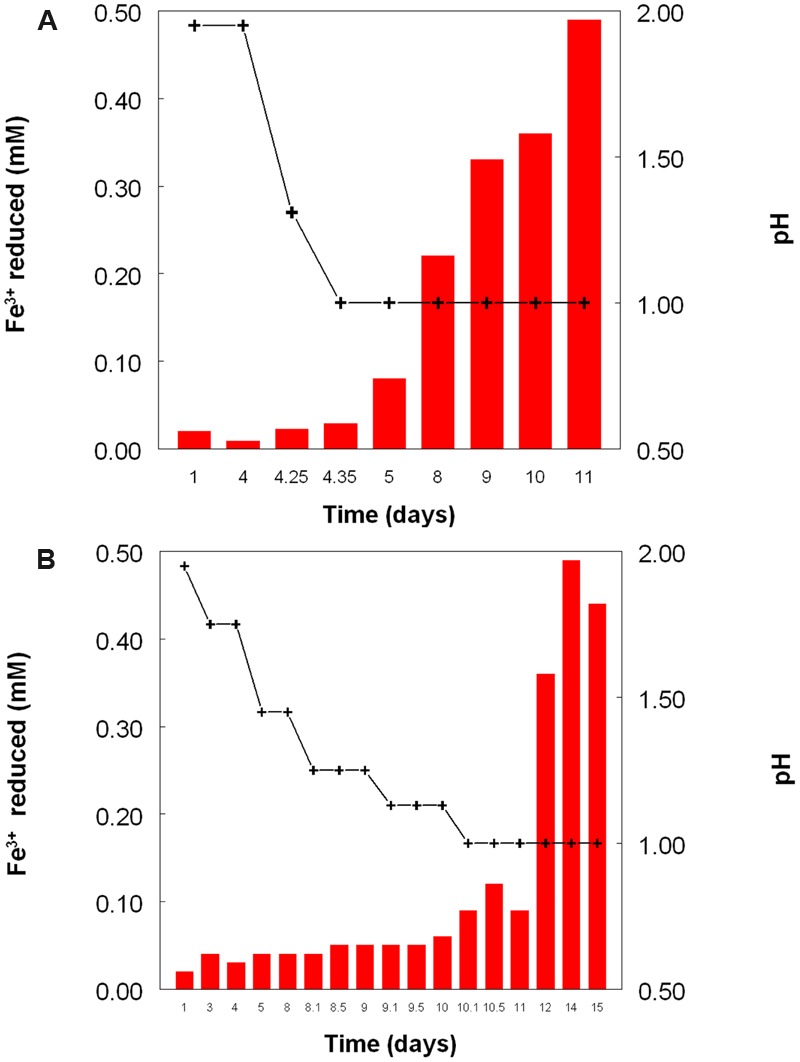
**Reduction of iron (III) (red bars) by samples of cultures of (A)** *At. caldus*^T^, and **(B)** *At. ferridurans*^T^ grown in bioreactors at varying pH. Stepwise decreases in bioreactor pH are depicted by the black line.

**FIGURE 3:**
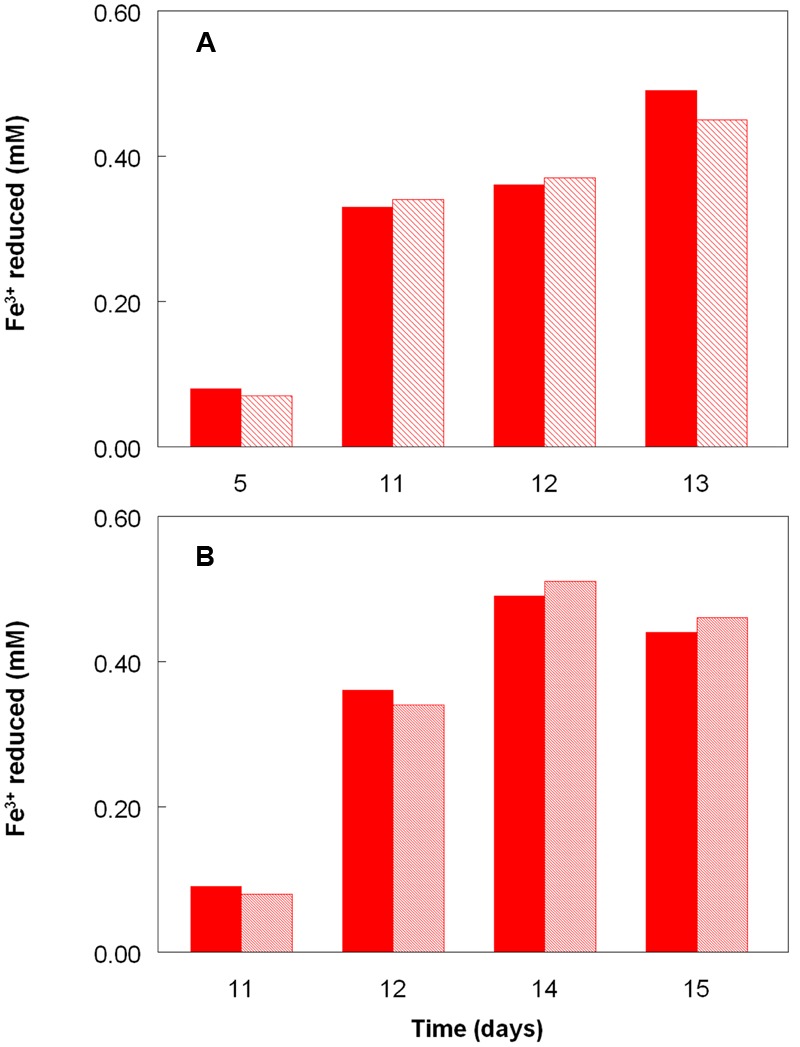
**Comparison of iron (III) reduction by bacteria-containing (solid bars) and cell-free culture liquors (hatched bars) of (A)** *At. caldus*^T^ and **(B)** *At. ferridurans*^T^, grown aerobically on elemental sulfur in bioreactors. The pH of both bioreactors at the times of sampling shown was 1.0.

**Figure 2** shows the potential of cultures of *At. caldus* and *At. ferridurans* to reduce iron (III), in samples removed from bioreactors at fixed pH values. Controlled acidification of the *At. caldus* bioreactor was carried out using fewer stages than for *At. ferridurans*, which is less tolerant of extremely low pH. While both *At. caldus* and *At. ferridurans* displayed some potential for reducing iron (III) in all samples removed from the bioreactors, this was far greater in those taken when the pH was 1.0 than at higher pH values. There was also a notable increase in iron (III) reduction potential during the 7 days period when the *At. caldus* bioreactor was maintained at pH 1.0. While numbers of planktonic cells of *At. caldus*, and, to a lesser extent, *At. ferridurans*, increased to some extent during the time course of this experiment, ensuring that these were ∼10^9^/mL at the start of the experiment minimized the impact of biomass size on the iron reduction data obtained. Comparison of iron (III) reduction by cell-free bioreactor samples and those containing bacteria (all from samples taken when both bioreactors were maintained at pH 1.0) showed little differences (**Figure 3**). All data shown in **Figures 2** and **3** are of iron (II) generated after 5 min incubation. Concentrations of iron (II) were usually significantly greater (about two-fold) after 2 h incubation but did not generally increase further with time, though again values were similar in both cell-free samples and those that contained bacteria with protracted incubation. No iron (III) reduction was observed in controls (i.e., basal salts/trace elements solutions adjusted to pH 1.0).

**FIGURE 4:**
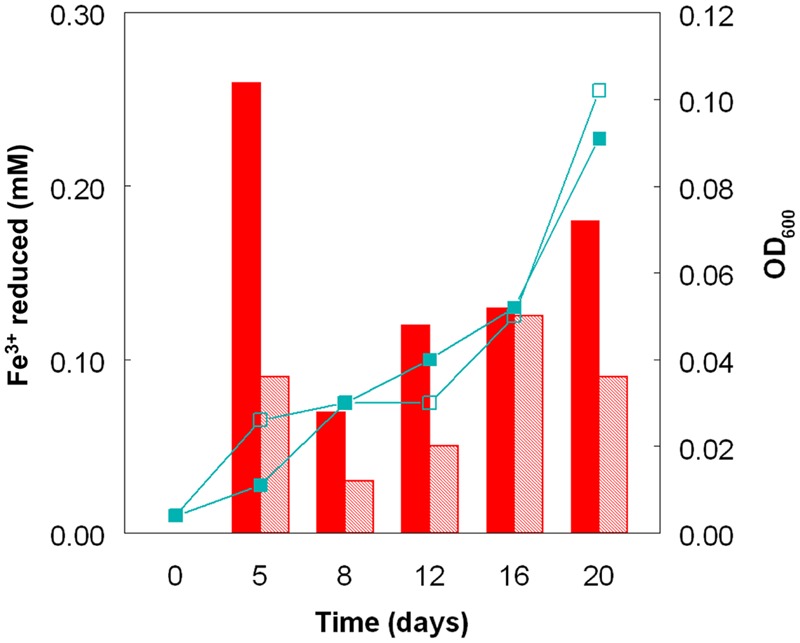
**Reduction of iron (III) by cell-free culture liquors of *At. caldus*^T^ grown aerobically on hydrogen at pH 1.0 (solid bars) and at pH 2.0 (hatched bars) to reduce iron (III).** The line graphs depict corresponding changes in culture optical densities (■, pH 1.0; □, pH 2.0).

Cultures of *At. caldus* grown aerobically on hydrogen at pH 1.0 and 2.0 showed similar growth rates and growth yields (**Figure 4**). The pH of these cultures did not alter much during incubation, since, in contrast to growth on iron (II) or reduced sulfur, aerobic oxidation of hydrogen neither generates nor consumes protons. As with sulfur-grown *At. caldus*, both bacteria-containing and cell-free samples from aerobic cultures of *At. caldus* grown aerobically on hydrogen were able to reduce iron (III). In most cases, cell-free samples from cultures grown at pH 1.0 were superior in this respect to those grown at pH 2.0, even though optical densities of these cultures were similar on each sampling occasion (**Figure 4**).

### Redox Transformations of Copper by Acidophilic Bacteria

Preliminary tests carried out with several species of acidophilic bacteria showed that, in every case, growth was inhibited by much smaller concentrations of copper (I) than has been reported elsewhere (and confirmed in the present study) for copper (II). The acidophiles tested displayed varying sensitivities to copper (I) which tended to parallel those to copper (II), apart from being far more acute [e.g., *L. ferrooxidans* was far more sensitive to both copper (I) and copper (II) than *L. ferriphilum*; **Table 2**]. In all subsequent experiments, care was taken to ensure that copper (I) concentrations present in culture media were within the range which allowed growth of the different species of acidophilic bacteria tested.

**Table 2:** Comparison of the relative toxicities of copper (I) and copper (II) to some species of acidophilic bacteria.

Bacterium	Copper (I)		Copper (II)	
	MGC	MIC	MGC	MIC
*At. ferrooxidans*^T^	8.5	11.5	400^1^	500^1^
*At. ferridurans*^T^	11.5	14	200^1^	300^1^
*L. ferrooxidans*^T^	1	2	10^2^	20^2^
*L. ferriphilum* MT63	8.5	11.5	200^2^	300^2^
*A. cryptum* SJH	2.4	4.4	15^3^	20^3^

*^1^Hedrich and Johnson (2013b); ^2^Galleguillos et al. (2009); ^3^Johnson (unpublished). Copper concentrations are mmoles/L. MGC, maximum concentration recorded for growth; MIC minimum inhibitory concentration.*

**FIGURE 5:**
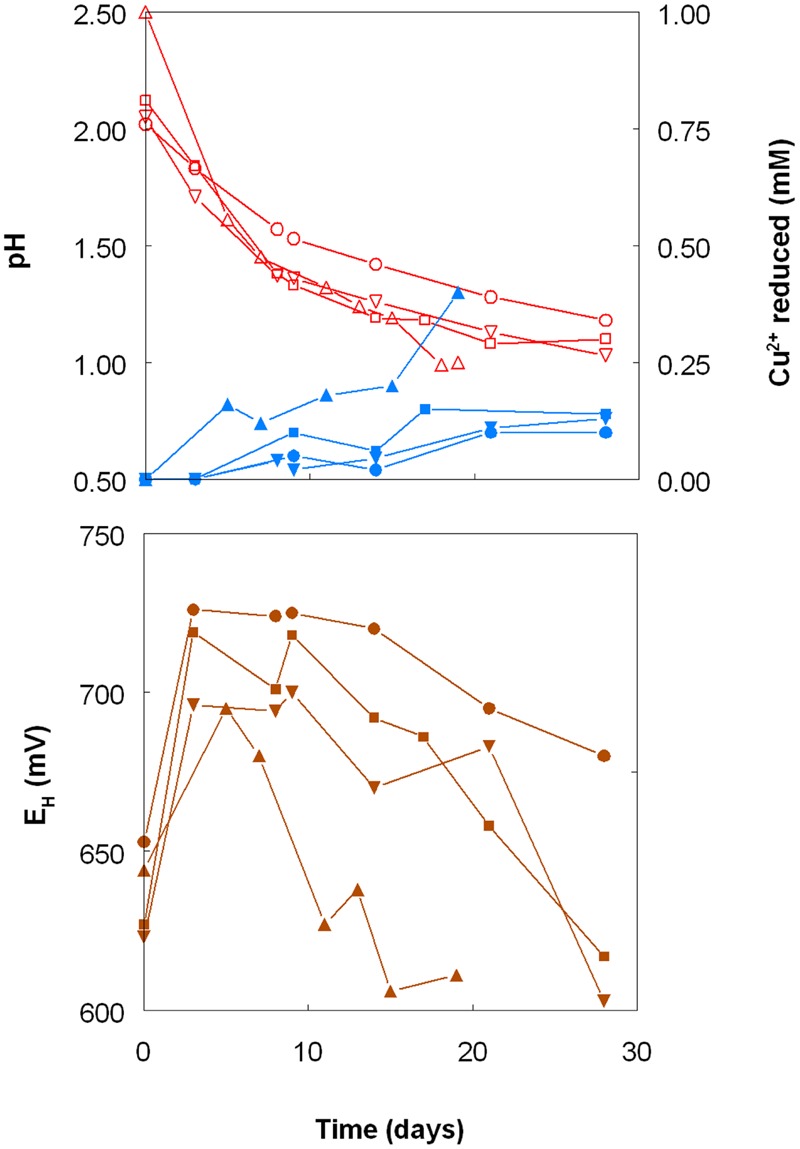
**Changes in pH values (red lines), copper (I) concentrations (blue lines) and redox potentials (*E*^H^; brown lines) in shake flask cultures of *Acidithiobacillus* spp. grown aerobically on elemental sulfur in the presence of 5 mM copper (II).** Key: (●, ○) *At. ferrooxidans*^T^; (■, □) *At. ferridurans*^T^; (▲, △) *At. caldus*^T^; (▼, ∇); *At. thiooxidans*^T^.

**FIGURE 6:**
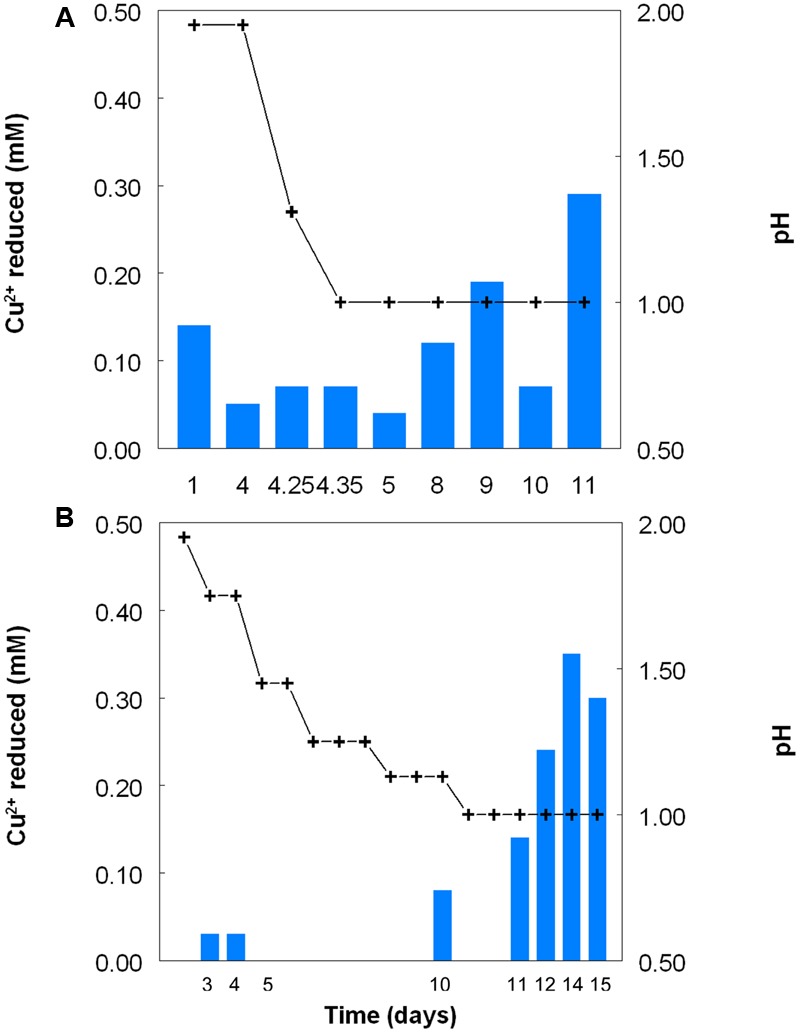
**Reduction of copper (II) (blue bars) by samples of cultures of (A)** *At. caldus*^T^, and **(B)** *At. ferridurans*^T^ grown in bioreactors at varying pH. Stepwise decreases in bioreactor pH are depicted by the black line.

Copper (I) accumulated in aerobic cultures of sulfur-grown *Acidithiobacillus* spp., though at slower rates and to lower final concentrations than those found with iron (III)-containing cultures (**Figure 5**). In contrast to cultures grown with iron, rates of sulfuric acid production were similar with all four *Acidithiobacillus* spp. tested, though more copper (I) was generated by *At. caldus* than by the other three species tested. Redox potentials in these cultures showed initial increases followed by continuous decreases in all cultures, and again the fluctuations in *E*^H^ values were more rapid in the case of *At. caldus* than with the other acidithiobacilli (**Figure 5**). Data in **Figure 6** show that samples taken from aerobic bioreactor cultures of *At. caldus* and *At. ferridurans* grown on elemental sulfur also reduced copper (II) to copper (I). In the case of *At. ferridurans*, copper (II) reduction potentials showed some degree of correlation to bioreactor pH values (*r*^2^ = 0.78) though there were insufficient data to conclude whether this was also the case for *At. caldus*. As with iron (III), reduction of copper (II) to copper (I) was found to be similar in both cell-free and bacteria-containing bioreactor samples (data not shown).

**FIGURE 7:**
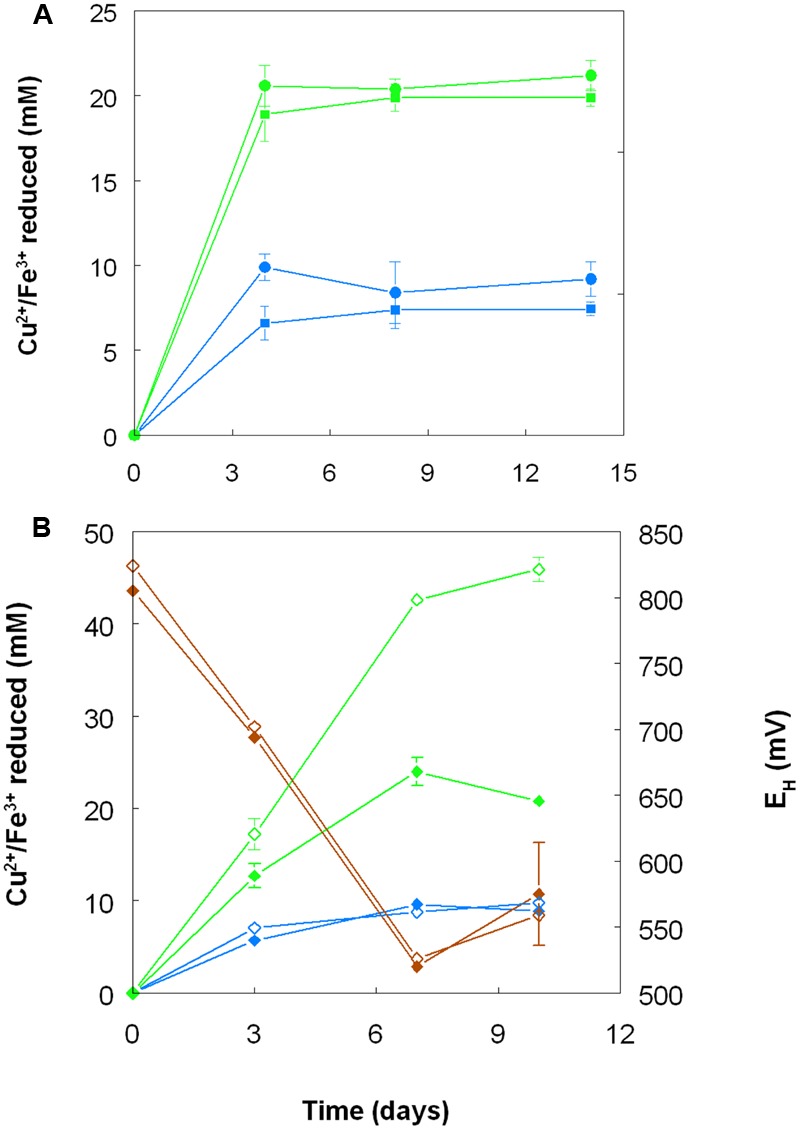
**Reduction of copper (II) (blue lines) and iron (III) (green lines), and changes in redox potentials (brown lines; data for *A. cryptum* only) in shake flask cultures of acidophilic bacteria grown under oxygen-limiting conditions. (A)** *At. ferrooxidans* (●) and *At. ferridurans* (■) grown under anoxic conditions with hydrogen as electron donor in liquid medium containing 20 mM of both copper (II) sulfate and iron (III) sulfate. **(B)** *A. cryptum* grown under micro-aerobic conditions with glucose as electron donor in liquid media containing 10 mM copper (II) sulfate and either 20 mM (filled symbols) or 50 mM (hollow symbols) iron (III) sulfate. No reduction of copper (II) was observed in cultures of all three acidophiles that did not initially contain iron (III).

When grown anaerobically on hydrogen with no iron (III) added, no growth or reduction of copper (II) to copper (I) was observed with all four *Acidithiobacillus* spp. (*At. ferrooxidans, At. ferridurans, At. ferrivorans*, and *At. caldus*) tested. However, when iron (III) was included in the medium, both it and copper (II) were reduced by *At. ferrooxidans* and *At. ferridurans*. **Figure 7A** shows that, in media containing initially 20 mM of both metals in their oxidized forms, virtually all of the iron was reduced, but only ∼50% of the copper, after 4 days of incubation. An identical trend of copper reduction occurring only in cultures that were amended with iron (III) was also observed with *A. cryptum* (micro-aerobic cultures only; growth and reduction of iron and copper was not observed under strictly anoxic conditions) though, in this case, ∼90% of the copper (II) present was reduced to copper (I). In cultures of this heterotrophic acidophile, redox potentials decreased by ∼300 mV during incubation and displayed minor reversions (increases of ∼50 mV) toward the end of the incubation period (**Figure 7B**).

**FIGURE 8:**
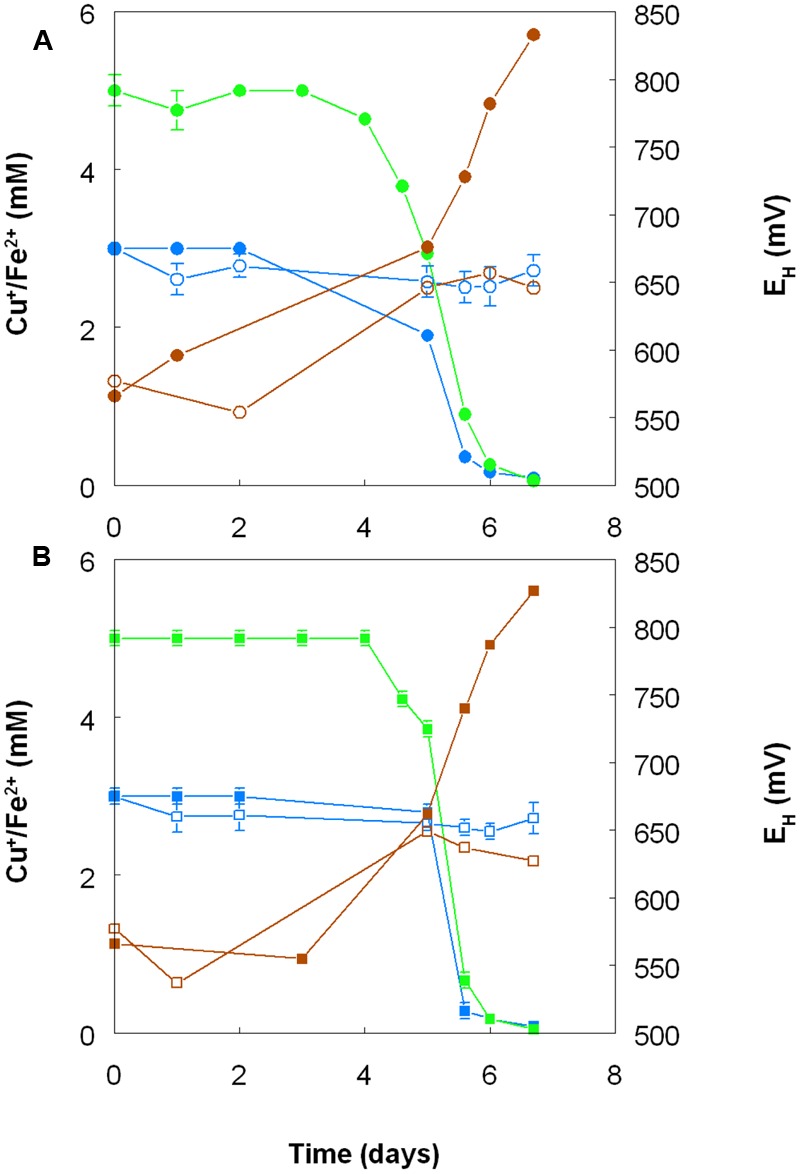
**Oxidation of copper (I) (blue lines) and iron (II) (green lines), and corresponding changes in redox potentials (brown lines) by cultures of (A)** *At. ferrooxidans* and **(B)** *At. ferridurans*, grown aerobically in cultures containing either both metals (filled symbols) or only copper (I) (hollow symbols). Error bars depict data ranges of replicate cultures.

Harvested biomass of *At. ferridurans* grown on hydrogen did not oxidize copper (I), in contrast to iron (II) which was oxidized (**Figure 7A**). No detectable changes in copper (I) concentrations were observed in non-inoculated controls over the same time. Likewise copper (I) was not oxidized in cultures of *At. ferrooxidans* or *At. ferridurans* that did not contain iron (II); small decreases in copper (I) concentrations in inoculated cultures over time were similar to those observed in non-inoculated controls (data not shown). In contrast, when iron (II) was also included in the culture medium, both it and copper (I) were oxidized, and redox potentials increased by >250 mV after a lag period of about 4 days in cultures of both iron-oxidizing *Acidithiobacillus* spp. (**Figure 8**).

### Redox Transformations of Chromium by Acidophilic Bacteria

Anionic chromium (VI) was shown to be far more toxic to the acidophilic bacteria tested than cationic chromium (III) (**Table 3**). No growth of any of the *Acidithiobacillus* spp. was observed in cultures (pH 2.0, with hydrogen as electron donor) to which chromium (VI) was added at 5 μM and above. In contrast, *A. cryptum* grew in the presence of 50 μM chromate at pH 2.5 (aerobically, with glucose as electron donor). In contrast, most of the iron-oxidizing autotrophic acidophiles grew in the presence of 50 mM chromium (III), though both *L. ferrooxidans* and the heterotroph *A. cryptum* were inhibited by this concentration of the metal but grew in the presence of 10 mM chromium (III) (**Table 3**).

**Table 3:** Comparison of the relative toxicities of chromium (III) and chromium (VI) to some species of acidophilic bacteria.

Bacterium	Chromium (III)		Chromium (VI)	
	MGC	MIC	MGC	MIC
*At. ferrooxidans*^T^	50	100	0^∗^	5^∗^
*At. ferridurans*^T^	100	>100	0^∗^	5^∗^
*At. ferrivorans* Peru6	100	>100	0^∗^	5^∗^
*L. ferrooxidans*^T^	50	100	nd	nd
*L. ferriphilum* MT63	10	50	nd	nd
*A. cryptum* SJH	10	50	50^∗∗^	100^∗∗^

*^∗^at pH 2.0; ^∗∗^at pH 2.5. Chromium (III) concentrations are mmoles/L, and chromium (VI) concentrations are μmoles/L. MGC, maximum concentration recorded for growth; MIC minimum inhibitory concentration; nd, not determined.*

No chromium (VI) was detected in chromium (III)-amended cultures of any of the five species of iron-oxidisers tested, irrespective of whether the growth media also initially contained iron (II). More positive redox potentials were recorded in (chromium-free) iron (II)-grown cultures of *Leptospirillum* spp. (+883 to +904 mV) than *Acidithiobacillus* spp. (+844 to +852 mV). However, while addition of chromium (III) caused the redox potential of oxidized *L. ferriphilum* cultures to fall by ∼30 mV, changes in *E*^H^ values were <10 mV in other cultures and, in all cases, no chromium (VI) was subsequently detected.

No growth or reduction of either chromium (VI) or iron (III) was found in cultures of the three *Acidithiobacillus* spp. incubated anaerobically with hydrogen as electron donor. However, all of the chromium (VI) added to anaerobic cultures of *At. ferridurans* in which the iron (III) present had been reduced to iron (II), was rapidly reduced to below detectable levels (∼10 μM). Tests of metal reduction carried out with cell suspensions of acidithiobacilli showed that, while no reduction of iron (III) occurred when these were sparged with N_2_, two of the three species (*At. ferridurans* and *At. ferrivorans*) reduced iron when hydrogen was provided. However, inclusion of 25 μM chromium (VI) to the cell suspensions completely inhibited reduction of iron (III), and no reduction of chromium was found to occur in iron-free cell suspensions.

Chromate reduction by *A. cryptum* showed some similarities but also some differences to results obtained with the acidithiobacilli. Concentrations of >25 μM chromium (VI) inhibited both growth and iron reduction under micro-aerobic conditions, even though cultures grew aerobically in the presence of 50 μM chromium (VI). All of the chromium (VI) added (up to 1 mM) to reduced (iron (II)-containing) cultures of *A. cryptum* was reduced, with concomitant generation of iron (III) (**Table 4**). Iron (III) was reduced by cell suspensions of *A. cryptum* maintained under micro-aerobic conditions only when glucose was added, while iron (III) reduction occurred in N_2_-sparged cell suspensions in both the presence and absence of added glucose. No reduction of chromium (VI) was observed in both micro-aerobic and anaerobic cell suspensions when this metal was added alone, but when both iron (III) and chromium (VI) were added to cell suspensions, both metals were reduced, again under micro-aerobic and anaerobic conditions (**Figure 10**).

**Table 4:** Changes in concentrations of iron (II) and chromium (VI) following addition of different concentrations of sodium chromate to a culture of *A. cryptum* SJH grown under micro-aerobic conditions, where all of the iron (III) present initially had been reduced to iron (II).

Chromium (VI) added (μmoles/L)	Fe^2+^(mmoles/L)	CrO_4_^2-^ (μmoles/L)
0	4.6	0
100	3.28	0
200	2.20	0
500	<0.05	0
1000	<0.05	40

### Redox Potentials of Iron, Copper, and Chromium in Acidic, Sulfate-Rich Liquors, and Solubility and Stability of Copper (I) in Acidic Iron (II) Solutions

**FIGURE 9:**
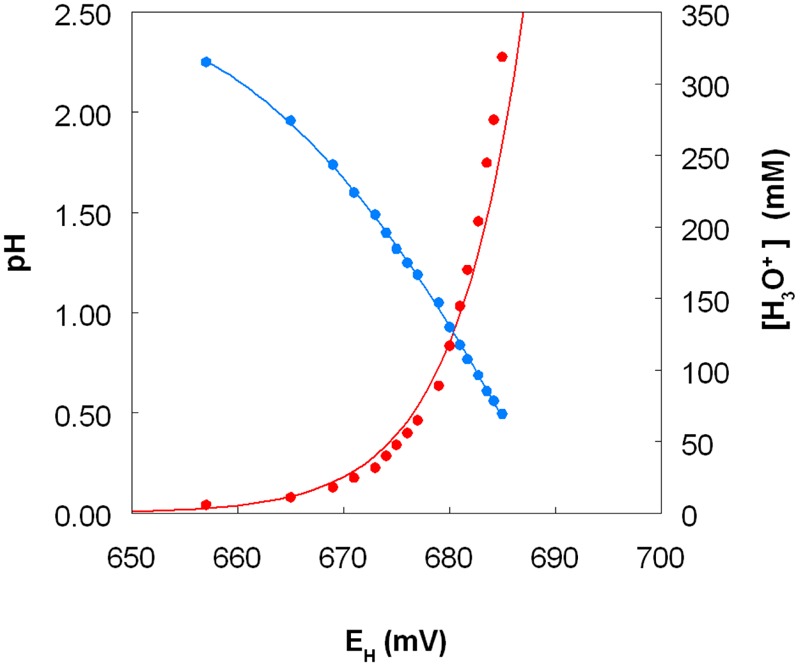
**Variation of the measured standard redox potential of the iron (II)/iron (III) couple measured in acidic sulfate-rich solutions as a function of pH (in blue) and as a function of calculated proton (hydronium ion) concentration (in red)**.

The standard redox potentials (*E*_H_^0^ values) of acidic, sulfate-containing solutions containing iron, copper or chromium present in both oxidized and reduced forms produced values which were, in each case, significantly different from those published for solutions where the metals are non-complexed. In the case of iron, the *E*_H_^0^ recorded at pH 2.0 was +663 mV, and this increased as the pH was lowered and decreased as pH was increased, beyond pH 2.0 (**Figure 9**, which shows *E*_H_^0^ values of the iron (II)/iron (III) couple as functions of both pH and calculated proton (hydronium ion) concentrations). Addition of 50 and 100 mM magnesium sulfate to the 10 mM iron (II) sulfate solution (at pH 2.25) caused the measured *E*_H_^0^ to decrease by 3 and 5 mV, respectively. The *E*_H_^0^ values of copper (I)/copper (II) sulfate solutions at pH 2.0, calculated using the Nernst equation using solutions where the molar ratios of the two ions varied between 1.0:4.0 and 2.9:2.1, were 548 ± 3 mV. Addition of 5 mM copper (II) sulfate to a solution of 10 mM iron (II) sulfate (both at pH 2.0) only resulted in minor changes in *E*^H^ values (2 to 3 mV). However, colorimetric analysis showed that concentrations of iron (II) were lowered by ∼1 mM as a consequence, inferring that ∼1 mM copper (II) had been reduced.

**FIGURE 10:**
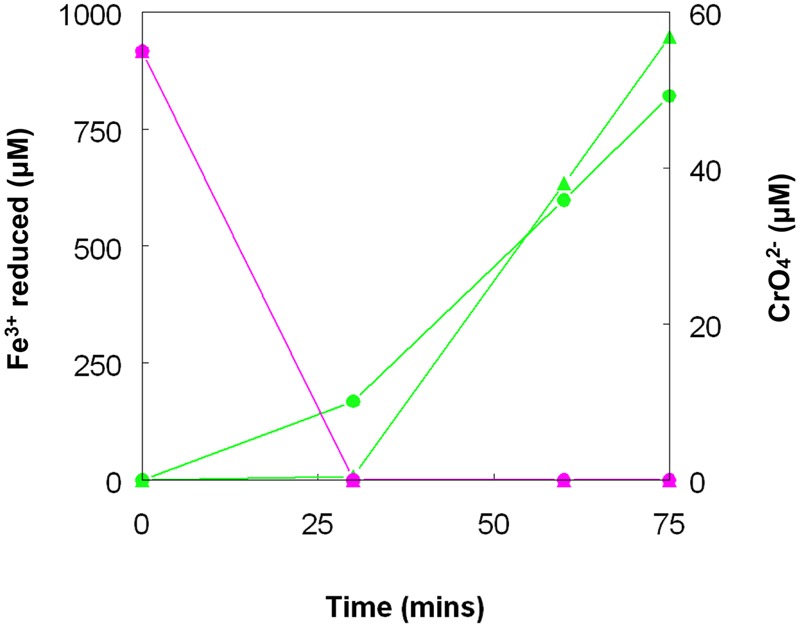
**Concomitant reduction of iron (III) (green lines) and chromium (VI) (magenta lines) by cell suspensions of *A. cryptum*.** Key: (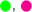) anaerobic suspensions; (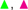) “micro-aerobic” suspensions. No reduction of chromium (VI) was observed when iron (III) was not added to cell suspensions.

Solubility and stability tests carried out with copper (I) chloride showed that, not only was this metal salt far more soluble in acidic iron (II) sulfate than in pure water adjusted to the same pH [15.2 mM in 1 M iron (II) sulfate compared to 2.7 mM in acidic (pH 2.0) water], but that copper (I) did not disproportionate (to copper (II) and elemental copper) over 24 h in the presence of either 100 mM or 1 M iron (II) sulfate, though there was clear evidence of disproportionation in both iron-free acidic water and 10 mM iron (II) sulfate solutions, as evidenced from the presence of soluble copper (II) and accumulation of films of elemental copper on the surfaces of the solutions (data not shown).

The measured *E*_H_^0^ of the chromium (III)/ chromium (VI) (chromate) couple at pH 2.43 was +844 mV. This varied with pH, increasing to +877 mV at pH 1.97, and to +895 mV at pH 1.78.

## Discussion

### Reduction of Iron (III) by *Acidithiobacillus* spp. in Aerobic Cultures

Shake flask experiments confirmed the observation by Sand (1989) that iron (III) could be reduced by *At. ferrooxidans*, grown aerobically on elemental sulfur at extremely low pH, but also showed that iron reduction also occurred in aerobic cultures of other *Acidithiobacillus* spp., including two that do not oxidise iron (II) (**Figures 1–4**). The pH at which rates of iron (III) reduction accelerated with both *At. ferrooxidans* and *At. ferridurans* (∼1.3) corresponded to the pH growth minima reported for both type strains (Hedrich and Johnson, 2013b). Since both species use iron (II) in preference to other electron donors (Yarzabal et al., 2004; Sandoval Ponce et al., 2012) it is likely that any iron (III) reduction that occurred within the growth pH range of these acidophiles would have been masked by re-oxidation of the iron (II) generated. This would not be the case with the moderate thermophile *At. caldus*, and it is interesting that iron (II) accumulated in cultures of this acidophile from close to the start of the experiment (**Figure 1**). A similar pattern was not, however, found with *At. thiooxidans*, a mesophilic species that also does not oxidize iron, where accumulation of iron (II) only became significant at and below pH 1.2.

The experiment carried out in pH-controlled bioreactors showed that the propensity of *At. caldus* to reduce iron (III) under aerobic conditions was also greatly affected by the pH of the growth medium (**Figure 2**). Samples taken from the bioreactor when maintained above pH 1.0, showed some, though relatively small, ability to reduce iron (III) rapidly, but this increased dramatically when the culture was held at pH 1.0. A similar scenario was observed with *At. ferridurans*. However, in both cases, bacterial cells did not have to be present for iron (III) reduction to occur (**Figure 3**), the implication being that the latter was mediated (for both species) by one or more soluble metabolites generated by the bacteria. Inorganic sulfur compounds, generated as waste products during growth on elemental sulfur (Steudel et al., 1987), have been implicated in mediating metal ion reduction by *Acidithiobacillus* spp. (Briand et al., 1996). However, hydrogen-grown *At. caldus* also displayed similar abilities to reduce iron, again irrespective of bacterial cells being present (**Figure 4**), confirming that sulfur intermediates were not solely (if at all) responsible for mediating aerobic iron (III) reduction by these acidophiles. It is also possible that redox-active biomolecules (e.g., cytochromes) are released into culture liquors during active growth or lysis of the acidophilic bacteria, and that these also mediate iron (III) reduction. Results from this work, showing that aerobic iron (III) reduction by *Acidithiobacillus* spp. appears not to be correlated with growth, support the observation by Hallberg et al. (2001) that species that do not oxidize iron (such as *At. caldus* and *At. thiooxidans*) cannot grow by respiring iron (III), unlike the iron-oxidizing acidithiobacilli.

### Reduction of Copper (II) and Toxicity of Copper (I) to Acidophilic Bacteria

Although tolerance of acidophilic bacteria to both iron (II) and iron (III) have been frequently reported in the literature, published copper tolerance data refer exclusively to copper (II). As found in the present study, copper (I) is much more toxic to these bacteria (**Table 2**), with MICs being generally an order of magnitude or more lower that those reported for copper (II). Copper occurs immediately above silver in the Periodic Table of elements, and it is interesting to note that monovalent silver (Ag^+^) is highly toxic to acidophilic bacteria, inhibiting growth when present in micro-molar concentrations (Tuovinen et al., 1985). Copper (I) is, however, both relatively insoluble and highly unstable in most aqueous solutions, where it disproportionates to copper (II) and elemental copper (2 Cu^+^ → Cu^2+^ + Cu^0^). Results from the current work have shown that, not only is the solubility of copper (I) chloride far greater in 1 M iron (II) sulfate (pH 2) than in water acidified to the same pH value, but that the stability of copper (I) is greatly enhanced by the presence of iron (II). This has important implications for acidic copper-rich waters, such as some acid mine drainage (AMD) streams and PLS.

**FIGURE 11:**
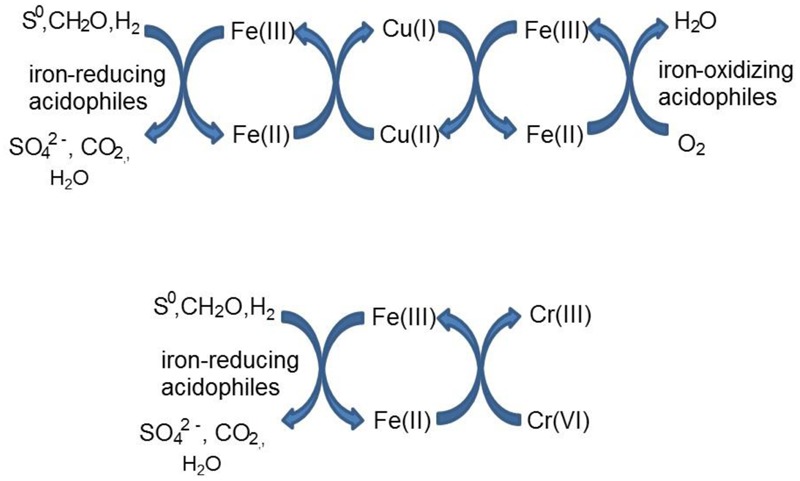
**Schematic representation of indirect redox transformation of copper and chromium mediated by acidophilic bacteria**.

*Acidithiobacillus* spp. grown aerobically on elemental sulfur also reduced copper (II), though far less copper (I) than iron (II) accumulated in shake flask cultures (**Figure 5**). The amounts of iron and copper reduced by bioreactor cultures of *At. caldus* and *At. ferridurans* tested off-line were more similar (**Figure 6**), and, as with iron, copper reduction was observed with cell-free culture liquors of both acidophiles, suggesting a common mechanism for iron and copper reduction by the acidithiobacilli grown under these conditions. When hydrogen (for autotrophic species) or glucose (for the heterotroph *A. cryptum*) was used as electron donor, the possibility that iron and copper reduction were both mediated by one or more sulfur metabolites was eliminated (**Figure 7**). While none of the *Acidithiobacillus* spp. tested, (*At. ferrooxidans, At. ferridurans, At. ferrivorans*, and *At. caldus*) were able to couple the oxidation of hydrogen to the dissimilatory reduction of copper (II) directly, at least two species (*At. ferrooxidans* and *At. ferridurans*) could do this indirectly using iron as an electron shuttle (**Figure 8**). A similar scenario was observed with *A. cryptum* grown under micro-aerobic conditions. The mechanism of copper (II) reduction therefore appeared to involve the reduction of iron (III) to iron (II) (which has been well documented in all three of these species), and the subsequent reduction of copper (II) by iron (II). This regenerates iron (III) which can again act as a direct electron acceptor for these acidophiles, allowing them to utilize copper (I) as an indirect electron acceptor (**Figure 11**).

The results of experiments carried out with both harvested biomass and active cultures of iron-oxidizing acidophiles showed that copper (I) did not act as a direct electron donor for these bacteria. However, it could act as an indirect donor in the presence of soluble iron, as demonstrated in cultures where iron (II) was included in the growth medium. Generation of iron (III) by the bacteria resulted in copper (I) being oxidized to copper (II), and the iron (II) so-formed being again oxidized to iron (III). This allowed the bacteria to harness the electron donor potential of copper (I) (**Figure 11**), even though they seemingly lack the enzymatic apparatus for oxidizing reduced copper directly.

### Redox Potentials of Iron and Copper Couples in Acidic, Sulfate-Rich Solutions

The fact that iron can both oxidize and reduce copper (and *vice versa*) might appear to be thermodynamically untenable. However, consideration of redox potentials of the iron (II)/iron (III) and copper (I)/copper (II) couples in acidic, sulfate-rich solutions resolves this apparent conundrum (**Figure 9**). The frequently quoted standard redox potential (*E*_H_^0^) of the iron (II)/iron (III) couple is +770 mV, and that of the copper (I)/copper (II) couple is ∼ +160 mV. However, these values apply to situations where both metals are present as soluble and non-complexed ions, and it is known for example that, in the presence of organic chelating agents such as citrate or haem, the *E*_H_^0^ value of the iron couple is considerably less positive than +770 mV. Most extremely acidic environments contain relatively small concentrations of dissolved organic carbon (<10 mg/L) and therefore organic chelation of iron is usually negligible. However, in acidic, sulfate-rich waters, such as AMD and PLS, iron (III) is complexed by both hydroxyl and sulfate anions, forming a range or cationic and anionic complexes [Fe(OH)^2+^, Fe(OH)_2_^+^, Fe(SO_4_)^+^, Fe(SO_4_)_2_^-^), the relative proportions of which vary with pH and sulfate concentrations (Welham et al., 2000). As a consequence, the *E*_H_^0^ of the iron (II)/iron (III) couple is significantly less than +770 mV in these waters (Johnson et al., 2012). In the present study, the measured *E*_H_^0^ was between +657 mV (at pH 2.25) and +685 mV (at pH 0.5) at a molar ratio of 2 iron/2.5 sulfate. Increasing the molar ratio more in favor of sulfate depressed the *E*_H_^0^ value (at pH 2.25) presumably by increasing the relative proportion of the Fe(SO_4_)_2_^-^ complex present, though the impact of sulfate did not appear to be as significant as that of solution pH. In contrast, the *E*_H_^0^ of the copper (I)/copper (II) couple in acidic, sulfate-rich solutions, calculated from measurements of *E*^H^ values of solutions containing known concentrations of both ions, was ∼400 mV more positive that the value commonly quoted. The consequence of the is that, although an iron (III)-rich liquor will always have a sufficiently positive redox potential to allow it to oxidize copper (I), an acidic iron (II) sulfate-rich solution can have a lower *E*^H^ value [+539 mV for a pH 2.0 solution containing 99% iron (II) and 1% iron (III) sulfate] than an acidic copper (II) sulfate-rich solution [+666 mV for a pH 2.0 solution containing 99% copper (II) and 1% copper (I) sulfate], values calculated using the Nernst equation and the *E*_H_^0^ values determined in the current experiments. Mixing the two solutions would therefore result in a partial reduction of copper (II) [and oxidation of iron (II)], as observed in the present work. Matocha et al. (2005) had previously reported that copper (II) could be reduced to copper (I) by iron (II) under abiotic conditions. The important difference between a static (chemical) situation and a dynamic (biological) scenario is that, in the latter, the continuous regeneration of iron (II) (as a consequence of dissimilatory iron reduction) would result in a much greater increase in copper (I) concentrations, as found in the experiments described herein.

### Toxicity and Redox Transformations of Chromium

Chromium (VI) was confirmed to be far more toxic to the extreme acidophiles examined than chromium (III) (**Table 3**). Metal oxyanions are known to be generally more toxic than metal cations to acidophilic bacteria due to the fact that, in contrast to neutrophiles, they have positive rather than negative membrane potentials (Norris and Ingledew, 1992). This could also help explain why *A. cryptum* was apparently more tolerant to chromium (VI) than the acidithiobacilli, as the former was grown at a higher pH than the latter which would result in its membrane potential being less positive.

The measured standard redox potential (*E*_H_^0^) of the chromium (III)/chromium (VI) couple was much more positive than either that of the iron or copper couples. This was not unexpected, though the values measured in the current work were less positive (+844 to +895 mV) than that generally quoted for the Cr_2_O_7_^2-^/Cr^3+^ couple (+1.33 V). Chromium (III), like iron (III), is readily complexed by inorganic anions, and the dominant form of the chromate anion varies with solution pH (existing mostly as HCrO_4_^-^ at pH 2.0, with undissociated H_2_CrO_4_ becoming increasingly relatively abundant as pH decreases; Tandon et al., 1984). There are no reports of direct biogenic oxidation of chromium (III) to chromium (VI), though this reaction can be mediated by manganese (III) or manganese (IV) minerals generated biologically (Lloyd et al., 2015). However, since the *E*_H_^0^ of the chromium (III)/chromium (VI) couple determined in acidic sulfate-containing liquors in the present study was actually slightly less positive than the maximum redox potentials of *Leptospirillum* spp. grown on iron (II), there was the possibility that some chromium (VI) may be generated indirectly by this iron-oxidiser. However, there was no evidence of chromium (VI) generation in these cultures either containing chromium (III) initially, or to which chromium (III) was added after iron oxidation had ceased. This was also the case with the *Acidithiobacillus* spp., though terminal *E*^H^ values of cultures of these iron-oxidisers were less positive, as recorded elsewhere (Rawlings et al., 1999), which is a reflection of their lower affinity for iron (II) and greater sensitivity to iron (III) than *Leptospirillum* spp. (Norris et al., 1988). The reason why no chromium (III) oxidation was observed even in cultures where *E*^H^ values exceeded +900 mV was possibly due to the amounts of chromium (VI) generated being below limits of detection, or because of the acute toxicity of chromium (VI) to iron-oxidizing acidophiles which would have limited the reaction.

As was the case with copper (II), there was no evidence to support the hypothesis that chromium (VI) can be reduced directly to chromium (III) by acidophilic bacteria, though this may occur indirectly *via* iron (II) generated under either anaerobic (*Acidithiobacillus* spp.) or micro-aerobic (*A. cryptum*) conditions (**Table 4**; **Figure 10**). The fact that chromium (VI) was reduced rapidly by cultures of *At. ferridurans* and *A. cryptum* that had already reduced iron (III) to iron (II) was not unexpected, as chromium (VI) is well known to be reduced by iron (II) in acidic solutions:

$${\mathrm{CrO}}_{4}{}^{2-}+3{\mathrm{Fe}}^{2}{}^{+}+8H^{+}\to {\mathrm{Cr}}^{3}{}^{+}+3{\mathrm{Fe}}^{3}{}^{+}+4H_{2}O.$$

Both metals were also reduced when they were added to cell suspensions in their oxidized forms, confirming that chromium (VI) and iron (III) reduction can be concurrent. This was demonstrated with *A. cryptum* only, as even 25 μM chromate was sufficient to totally inhibit iron reduction by the acidithiobacilli. The inability of *A. cryptum* to reduce chromium (VI) in the absence of iron contradicts the earlier findings of Cummings et al. (2007) who reported that cell suspensions of *A. cryptum* strain JF-5 could reduce chromium (VI) directly, whether or not an electron donor (glucose) was provided. They also suggested that the reaction was enzymatic. However, the rate of chromium (VI) reduction reported by Cummings et al. was very slow, requiring 10 h to reduce concentrations from 50 μM to about 10 μM (though this was greatly accelerated when iron was added), compared to near complete indirect reduction of 50 μM chromium (VI) within 30 min found in the present study. Possible reasons for the discrepancy in the results of the two studies include: (i) strain variation (though *A. cryptum* strains SJH and JF-5 share >99.5% similarity of their 16S rRNA genes); (ii) growth media and carry over of organic materials [Cummings et al. (2007) grew their strain on a tryptone soya broth/vitamins liquid medium and some complex organic compounds are known to reduce chromium (VI), while *A. cryptum* SJH was grown in glucose/minimal salts, which would have eliminated this possibility]; (iii) the presence of iron (II) or iron (III) (which would be reduced) in cell suspensions in the earlier study, as only trace amounts (micro-molar concentrations) of iron (II) are necessary to reduce equivalent concentrations of chromium (VI). Masaki et al. (2015) also included tryptone soya broth in the media they used to cultivate *Acidocella aromatica* (which was not necessary, as this acidophile cannot utilize the organic components present in this material) and, interestingly, found that chromium (VI) was reduced even in aerobic cultures. The results of the present study lead us to conclude that, while chromium (VI) can be reduced by acidophilic prokaryotes, this is mediated indirectly by species that reduce iron (III) to iron (II) (**Figure 11**), and that evidence for direct reduction requires re-evaluation and validation.

### Implications for Biotechnology and the Biosphere

The results of the current study have widespread implications for established and emerging biotechnologies that utilize acidophilic microorganisms. For example, reductive dissolution has been proposed as a means of extracting base metals such as nickel and cobalt from oxidized ores, such as laterites (Johnson et al., 2013). The process was originally demonstrated using anaerobic bioreactors containing acidophilic bacteria that couple the oxidation of elemental sulfur to the reduction of iron (III), though more recently Marrero et al. (2015) reported that reductive dissolution of laterite tailings occurred in aerobic bioreactors containing *At. thiooxidans* and *At. ferrooxidans* at extremely low pH. Data from the current study, showing that iron (III) was reduced by several species of *Acidithiobacillus* at pH values where re-oxidation of the iron (II) produced by *At. ferrooxidans* does not occur, support their findings, though it is worth noting that iron (II) can be oxidized at pH < 1.0 by more acid-tolerant acidophiles (*Leptospirillum* and *Ferroplasma* spp.) which would obviate mineral dissolution *via* a reductive mechanism. The findings that not only copper (II) can be reduced indirectly by iron-reducing acidophiles but also that copper (I) is a far more toxic form of this metal to biomining bacteria, should be taken into account when managing PLS produced by heap bioleaching at copper mines, where mineral-oxidizing prokaryotes have a critical role in metal extraction. Finally, indirect reduction of highly bio-toxic chromium (VI) to the less harmful chromium (III) cation could be used to remediate Cr-contaminated wastewaters. The advantage of using acidophiles in this process is that the chromium (III) generated would be maintained in solution rather than precipitated within a bioreactor, thereby facilitating its downstream recovery.

Apart from the industrial connotations, indirect oxido-reduction of copper and, to a lesser extent, reduction of chromium (VI) has potential implications for lithotrophic life on our own planet, and possibly beyond. The implication is that copper (I) minerals, such as chalcocite (Cu_2_S) could act as sources of energy, not only for acidophilic bacteria such as *Acidithiobacillus* spp. that can oxidize reduced sulfur (as reported by Nielsen and Beck, 1972), but also for iron-oxidisers, such as *Leptospirillum* spp. and *Ferrimicrobium acidiphilum*, that only oxidize iron (II), allowing them to exploit low pH environments that are rich in reduced copper but contain relatively little iron. The reverse reaction, whereby microbial growth could be supported by indirect dissimilatory reduction of copper (II), could also be significant in copper-rich environments. Both processes provide tantalizing scenarios of how lithotrophic prokaryotes might thrive in geological niches previously considered as both hostile and unimportant for life.

## Author Contributions

DBJ: experimental work, writing manuscript, preparing Figures and Tables. SH: conceptual discussions, editing of manuscript. EP: experimental work, editing of manuscript.

## Conflict of Interest Statement

The authors declare that the research was conducted in the absence of any commercial or financial relationships that could be construed as a potential conflict of interest.
